# Microleakage of CEM Cement and ProRoot MTA as Furcal Perforation Repair Materials in Primary Teeth

**Published:** 2013-10-07

**Authors:** Roza Haghgoo, Sara Arfa, Saeed Asgary

**Affiliations:** a Department of Pediatric Dentistry, Dental School, Shahed University, Tehran, Iran;; b Dental Research Center, Research Institute of Dental Sciences, Shahid Beheshti University of Medical Sciences, Tehran, Iran;; c Iranian Center for Endodontic Research, Research Institute of Dental Sciences, Shahid Beheshti University of Medical Sciences, Tehran, Iran

**Keywords:** Calcium Enriched Mixture, CEM Cement, Furcal Perforation, Microleakage, ProRoot MTA, Sealing Ability

## Abstract

**Introduction:**

Iatrogenic furcal perforation is a procedural accident in endodontic treatments of primary/permanent teeth; prognosis may be favorable if a complete seal with biomaterial is immediately established. The purpose of this *in vitro* study was to evaluate microleakage of calcium enriched mixture (CEM) cement and ProRoot mineral trioxide aggregate (MTA) for sealing primary molar furcal perforations.

**Materials and Methods:**

This study was conducted on 38 extracted human primary molars. Furcation perforations were created in the pulp chamber floor. The teeth were divided randomly in two experimental groups (*n*=17) and two positive and negative controls (*n*=2). Perforations were then repaired with biomaterials. After 72 h, the teeth were submerged in 2% fuchsin dye solution for 24h. The samples were sectioned longitudinally and evaluated for dye leakage. Data analyzed statistically using ANOVA test.

**Results:**

The negative and positive controls behaved as expected. Dye microleakage was observed in all experimental samples; however, there was no statistically significant difference between the microleakage of MTA (4.411±2.042 mm) and CEM (3.647±1.040 mm) groups (*P*>0.05).

**Conclusion:**

Based on the findings of this *in vitro* study, CEM and tooth-colored ProRoot MTA have similar sealing ability for furcal perforation repair of primary molar teeth.

## 1. Introduction

Pathological/mechanical perforation can be defined as a communication between the root canal and external root surface or periodontal ligament. Furcal perforation of molar teeth can lead to tooth loss; however, if such perforations are immediately diagnosed and sealed with a biocompatible material, the prognosis is usually good [1, 2]. Perforations can be managed surgically or nonsurgically. In nonsurgical treatment, perforations should be repaired swiftly with a biomaterial to prevent bacterial contamination and communication between perforation site and gingival sulcus [3].

The ideal material for sealing perforations should be biocompatible, non-toxic, bactericidal or bacteriostatic, radiopaque, non-absorbent, and good seal. Moreover, they should possess the ability to induce osteogenesis and cementogenesis [4-6]. Several materials have been suggested for perforation repair such as amalgam, calcium hydroxide, reinforced zinc oxide eugenol cements, mineral trioxide aggregate (MTA) and calcium enriched mixture (CEM) cemen [7-9].

MTA biomaterial was introduced in 1993 by Torabinejad [10]. It has alkaline pH after setting and hardens in ~4 h; basic compositions of MTA are tricalcium and dicalcium silicate, tricalcium aluminate, tetracalcium aluminoferrite, calcium sulfate and bismuth oxide [11]. MTA can induce cementum deposition when used to seal perforations [12]; it has superior sealing ability compared to other restorative materials, when used for repairing perforations [13].

Calcium enriched mixture (CEM) cement is tooth-colored and hydrophilic endodontic biomaterial [14]; it can set in wet environment and therefore is ideal as a perforation repair biomaterial [9]. Electron probe microanalysis (EPMA) results showed that major elements in CEM cement are CaO, P_2_O_5_, SO_3_, and SiO2; moreover, this cement has similar pH, decreased working time,

**Table 1.** Statistical indices for leakage in experimental groups in mm

Group	n	Mean (SD)	Max.	Min.
**MTA**	17	4.411 (20.42)	7.0	1.0
**CEM**	17	3.647 (10.40)	5.0	1.0

increased flow and film thickness compared to MTA [14]. Recent randomized controlled trials have revealed successful outcomes following vital pulp therapies of primary molar teeth using this novel biomaterial [15, 16]. A histological and CBCT evaluation of a pulpotomized human primary molar tooth demonstrated complete dentin bridge formation beneath the CEM [17]. A recent case report showed favorable treatment outcomes after one-month delayed CEM furcal perforation repair of a symptomatic permanent molar associated with a large furcal lesion. The 2-year findings reported absence of sign/symptoms and healing of furcal lesion [18]. A more recent clinical study demonstrated that root perforations repair with CEM in primary molars with extensive inflammatory root resorption/perforations associated with periodontal lesions resulted in complete healing [19]. Animal studies evaluating tissue responses to MTA and CEM in furcal perforation repair sites, demonstrated that both MTA and CEM have similar and favorable response in repair of furcal perforation by cementogenesis over the biomaterials [9]. Many studies reported that CEM is a biocompatible material with low cytotoxicity, antibacterial properties, good sealing and ability for strengthening immature roots [20-23]. Clinical applications of the biomaterial including root-end filling, pulp capping, pulpotomy of primary and permanent teeth, apexification of open apex teeth, repair of internal and external root resorption, as well as root canal filling [15, 16, 24-29].

The aim of this *in vitro* study was to compare the sealing ability of CEM cement and MTA when used to repair the furcal perforation of primary molars using a dye leakage model.

## 2. Material and Methods

Thirty-eight extracted human maxillary/mandibular primary molars were used in this study. The teeth had minimal caries and normal furcation. The teeth were kept in 5% sodium hypochlorite (Shamin Co., Tehran, Iran) for 30 min. They were then washed with tap water and stored in normal saline. A standard access cavity was prepared in each tooth using a diamond bur (#05, D&Z Co., Wies Baden, Germany) in high speed handpiece with water spray. The teeth were decoronated ~4 mm above the CEJ and then roots were horizontally cut off in the mid-root. Root canal orifices and the apical end of each root were etched in all groups with 37% phosphoric acid gel (Kimia, Chemdent, Iran) for 30s. Tetric N-Bond adhesive (Total-Etch, Ivoclar Vivadent, CA, USA) was then applied in two coats and photo polymerized with an LED source. A light-cured flowable resin composite (DiaDent, Diafil, Korea) was then used to fill the root canal orifices and the apical end of roots.

A perforation was made with a size 010 round bur (D&Z Co., Wies Baden, Germany) in a high-speed water cooled handpiece in the centre of the pulp chamber floor. The bur was replaced after every five perforations. The width of all perforations was similar, but the length of the perforation depended on the dentine-cementum thickness from pulp chamber to furcation area. The samples were randomly divided into two experimental groups (*n*=17) of white MTA (ProRoot, Dentsply, Tulsa, OK, USA) and CEM cement (BioniqueDent, Tehran, Iran). In the positive control two teeth were perforated but not repaired; in 2 negative controls the perforations covered with two layers of nail varnish.

The teeth were placed in soft sponge. MTA and CEM were mixed according to the manufacturer’s instruction to produce a homogeneous paste. Subsequently, the biomaterials were placed on the perforation area by a MTA carrier and gently compacted with cotton pellets. On the completion of repair procedure, the materials were covered with moistened cotton pellets and the access cavity sealed with Cavit (3M ESPE, Seefeld, Germany). The teeth were placed in an incubator at 37°C and 95% humidity for 72 h.

The teeth were then covered with two layers of nail polish except ~1 mm around the perforated area so that the dye would only penetrate through the furcation area. All the teeth were immersed in 2% basic fuchsin (Sigma-Aldrich Co., LLC, USA) for 24 h; they were removed from the solution, rinsed and then mounted in transparent acrylic. Samples were sectioned mesiodistally parallel to long axis of the teeth and linear dye penetration was measured on each wall from the apical end of the perforation to the pulp chamber floor using a stereomicroscope (20× Mag.; Carton optimal industries Ltd., SCW-E, Thailand). The microleakage recorded and analyzed statistically by ANOVA test.

## 3. Results

Maximum dye penetration was observed in positive control group; the samples showed complete dye penetration. In contrast, negative control group did not show any dye penetration. The mean and standard deviation for dye penetration in experimental groups are shown in Table 1. There was no statistically significant difference in dye penetration between MTA and CEM cement. 

## 4. Discussion

The success of furcal perforation repair is dependent on an effective seal between the inner and outer tooth environment. This can be achieved with a suitable endodontic material. Repair materials should stop the microleakage of microbial irritants from the root canal into the periodontal tissues. Various models are currently used to evaluate the leakage. Dye penetration technique is one of the most common methods due to its easy performance [30]. Several types of dyes have been suggested *i.e*. Indian ink, fuchsin, methylene blue, silver nitrate and rhodamine B. The dye’s pH, chemical reactions and molecular size affect the degree of dye leakage [31]. Methylene blue is a low-priced dye which has been commonly utilized; however, researchers demonstrated that optical density value of methylene blue decreases with MTA, which can cause underestimation in leakage studies [32]. The primary cause is the acidity of the methylene blue dye; therefore, alkaline dyes are more suitable for dye leakage studies on MTA. As studies have not reported any signs of dissolution of basic fuchsin by MTA, we employed this dye.

Our study is the first investigation that compares sealing ability of CEM and MTA in repair of furcal perforation in primary molars. MTA and CEM are hydrophilic endodontic cements; this feature facilitates wetting of dentin, allows access of cement within gap/spaces associated with the perforation walls and helps the entrance of small cement particles into dentinal tubules. Furthermore, MTA and CEM in contrast with other dental materials exhibit slight expansion after setting [14] and so provide enhanced adaptation of the biomaterials to the perforation walls. In addition, MTA and CEM form hydroxyapatite and provide an improved seal at the interface of biomaterials and dentin walls as well as the filling material [33, 34]. Various studies have showed excellent sealing ability for ProRoot/MTA in repair of perforations; they verified its superiority in contrast to other dental materials [7, 35-37]. However, several studies compared the sealing ability of CEM with MTA in various applications with often comparable results [23, 38-42].

To obtain clinical success, the perforation repair materials should result in formation of new bone, PDL and cementum in an ideal state. Previous studies revealed that cementogenesis is a key factor in dentoalveolar regeneration which was induced by MTA and CEM in canine models [9, 43]. The occurrence of cementogenesis surrounding the perforation repair biomaterials is ideal. Formed cementum is a biologic barrier against the spread of microbial irritants within the root canal system [44]. Case studies regarding the furcal perforation repair using MTA or CEM in permanent teeth showed that these biomaterials are capable of complete regeneration of adjacent dentoalveolar tissues [18, 45]. Recent reports stated that CEM and MTA may be suitable for closing the communication between the pulp chamber and the underlying periodontal tissues in primary teeth [19].

## 5. Conclusion

Based on the results of this study, CEM may be used in repair of furcal perforations of primary molar teeth. Further clinical evaluations are required to confirm this finding.
